# Postnatal Testosterone Concentrations and Male Social Development

**DOI:** 10.3389/fendo.2014.00015

**Published:** 2014-02-21

**Authors:** Gerianne M. Alexander

**Affiliations:** ^1^Department of Psychology, Texas A&M University, College Station, TX, USA

**Keywords:** postnatal testosterone, social development, infancy, sex differences, autism spectrum disorder

## Abstract

Converging evidence from over 40 years of behavioral research indicates that higher testicular androgens in prenatal life and at puberty contribute to the masculinization of human behavior. However, the behavioral significance of the transient activation of the hypothalamic–pituitary–gonadal (HPG) axis in early postnatal life remains largely unknown. Although early research on non-human primates indicated that suppression of the postnatal surge in testicular androgens had no measurable effects on the later expression of the male behavioral phenotype, recent research from our laboratory suggests that postnatal testosterone concentrations influence male infant preferences for larger social groups and temperament characteristics associated with the later development of aggression. In later assessment of gender-linked behavior in the second year of life, concentrations of testosterone at 3–4 months of age were unrelated to toy choices and activity levels during toy play. However, higher concentrations of testosterone predicted less vocalization in toddlers and higher parental ratings on an established screening measure for autism spectrum disorder. These findings suggest a role of the transient activation of the HPG axis in the development of typical and atypical male social relations and suggest that it may be useful in future research on the exaggerated rise in testosterone secretion in preterm infants or exposure to hormone disruptors in early postnatal life to include assessment of gender-relevant behavioral outcomes, including childhood disorders with sex-biased prevalence rates.

Testicular androgens have a central role in human male development. In prenatal life, increased testicular androgens around 4–6 weeks gestation masculinize the genitalia and initiate the sexual differentiation of the brain through hormonally dependent, sex-specific changes in the ultrastructure of the developing central nervous system (e.g., cell proliferation, cell death, patterns of cell migration, dendritic branching) (1). After a childhood of relative testicular quiescence, rising androgens at puberty activate the development of secondary sex characteristics and the maturation of the reproductive system (2). Converging evidence from approximately 40 years of research on human development indicates these hormonal changes also contribute to the masculinization of behavior (3, 4). However, the behavioral significance of another related surge in testicular androgens that results from a transient activation of the hypothalamic–pituitary–gonadal (HPG) axis in early postnatal life remains largely unexplored. It is significant, therefore, that recent findings from our laboratory suggest the postnatal endocrine surge may contribute to gender-linked typical and atypical social development.

## Postnatal Testosterone Surge and Early Theoretical Challenges to Behavioral Investigation

In infant boys, serum testosterone (T) concentrations that are gonadal in origin increase to pubertal concentrations between 1 and 3 months of age and fall to prepubertal values around 6 months of age (5–7). Salivary measures of bioavailable T in male neonates show concentrations well below those in men (8–10). Nonetheless, the results of a mammalian cell bio-assay provide direct evidence of biologically active androgens in male infants at 3 months of age (11) consistent with other findings that the postnatal surge contributes to the normal growth of male-external genitalia and sperm production (12). In a large prospective study of male infants, for example, higher concentrations of free T at 3 months of age predicted greater penile growth across the first 3 years of development (13).

In view of evidence indicating the postnatal endocrine surge is necessary to establish normal adult male reproductive function, a reasonable question is whether it is also necessary to establish male behaviors that facilitate reproductive success. An early perceptive commentary (14) suggested that the transient activation of the HPG axis may contribute to the sexual differentiation of primate behavior and expressed related concerns that the exaggerated rise in T secretion in preterm infants (15) or exposure to hormone disruptors in early postnatal life (16) may have adverse consequences for male primate reproductive development. Yet, 15 years passed before tests of the postnatal developmental programing hypothesis appeared in the human hormone-behavior literature.

The long delay in examining the postnatal developmental programing hypothesis in humans is understandable given the paucity of supporting evidence from studies in non-human primates. Investigations using a variety of mammalian species had established that higher androgen concentrations in prenatal life promoted a male behavioral phenotype in juveniles (17), including higher frequencies of rough-and-tumble play and mounting behavior in non-human primates (18). In contrast, independent laboratories reported that transient suppression of T in early postnatal life (19–21) had no measurable effects on the later expression of primate behaviors described as the “fundamental sexually dimorphic behavioral characteristics of the male” [(20), p. 334]. Suppression of T in males was reported in one investigation to decrease male juvenile independence from the mother, as measured by increased (i.e., more female-typical) proximity seeking relative to controls (20). However, whereas the prenatal period as a critical time in the sexual differentiation of primate behavior was supported by extensive experimental evidence, a similar role for the postnatal period was not.

Additionally, pioneering researchers of human hormone–behavior relations argued that the effects of androgens would be most apparent on behaviors showing large sex differences in expression, for example play preferences in childhood (17). Research applying this reasoning established that girls exposed to higher concentrations of prenatal androgens because of congenital adrenal hyperplasia (CAH) showed masculinized play relative to unaffected relatives, as measured by their stronger preference for toys typically preferred by boys (e.g., vehicles, balls) and weaker preference for toys typically preferred by girls (e.g., dolls) (22). Subsequently, masculinizing effects were found to extend to other behaviors showing large sex differences: girls with CAH showed enhanced targeting ability (23), increased levels of aggression (24, 25), and reported elevated activity levels (25).

Thus, studies of hormone–behavior relations in early postnatal life were likely further delayed because infants lack the physical and cognitive maturity to express known androgen-sensitive behaviors, such as aggression or toy play. In addition, few investigations specifically examined sex differences in infant behavior (26, 27). Limited research targeting relevant variables, such as activity levels (28), found small sex differences in infant behavior suggestive of small or absent hormone effects at this time in development. Finally, early gender socialization, apparent in infants’ gender-specific clothes and toys (29), raised the possibility that small sex differences in infancy may be attributable to the differential treatment of males and females and care-giver expectations of gender-congruent behavior. Supporting the role of gender expectancies, researchers reported that the assignment of a gender label resulted in differences in the perceived emotional responsiveness of an infant, such that a negative emotion was labeled “anger” if the infant was thought to be a boy, and “fear” if the infant was thought to be a girl (30). Significantly, although these findings have been cited over 130 times, the reported effects of gender labels on adult perceptions of infant behavior were not replicable (31, 32). Moreover, adults’ subjective perceptions of infant girls as smaller softer, and finer featured than boys (33) are supported by objective measures showing that compared to boys at birth, girls have higher length-to-weight ratios (34), a less prominent chin, and anteriorly narrower dental arcade (35).

Although other research has shown that gender labels influence adult response to infants (36, 37), we suggest that the differential treatment of male and female infants results from a bi-directional relationship between biological and social factors evident from birth: infants categorized as male and female on the basis of their genitalia, differ in other ways that support expectations based on gender labels. For instance, consistent with known sex differences in adult personality, meta-analyses of gender differences in infant temperament found that compared to girls, boys are less able to inhibit responses, and show less sensitivity to environmental changes, less fearfulness, and higher activity levels (38). Boys shortly after birth show stronger visual preferences for a mechanical mobile than for a face (39) and infant boys compared to girls show less orientation to a face or voice (39, 40) and shorter eye contact with an experimenter (41). Similar to the sex difference in adult emotional processing, infant boys also show less discrimination of emotional expression than their female counterparts (42) and appear to show a weaker propensity to empathic reactions, as indicated by shorter crying responses to recordings of a cry from a female infant (43, 44). Overall, male infants compared to female infants appear to differ in their responsiveness to stimuli with cues and characteristics associated with an animate form. Greater responsiveness to such cues in females may support social behaviors that enhance their reproductive success, such as interest in infants and “tend and befriend” responses to threat (45). As such, sex differences in infancy may indicate a biological preparedness for gender roles that for full expression requires the subsequent coupling of these early sex differences with social experiences imposed by contemporary gender socialization (46, 47).

## Postnatal Salivary Testosterone and the Emergence of Gender-Linked Social Relations

Small sex differences in infant temperament may represent essential structures supporting the development of adult sex differences in socio-emotional behavior (27) and recent evidence suggests the transient activation of the HPG axis may influence this process. Language researchers examining brain functional asymmetries first documented an association between serum T concentrations in infants at 4 weeks of age and developing language systems (48). In that research, female infants showed an advantage over males on a phoneme discrimination task that elicited bilateral activation of brain hemispheres, as measured by EEG. Male infants with low postnatal serum T compared to high T male infants showed phoneme discrimination with only left hemisphere activation. In contrast, male infants with higher postnatal serum T showed no discrimination effect, suggesting a relationship between postnatal androgen concentrations and developing brain systems that may contribute to later sex differences in language processing.

At the same time, studies on gender-linked toy preferences in infancy were ongoing in our lab using eye-tracking technology to measure infant interests (8, 46). Although toys are cultural artifacts linked to domestic and non-domestic activities that define traditional gender roles (49), biological influences on children’s toy choices are indicated by similar sex-linked object preferences in two non-human primate species (50, 51) and findings noted above that higher prenatal androgen concentrations in girls are associated with masculinized toy preferences (22). We examined toy preferences in 30 infants at 3–8 months of age by measuring the number of visual fixations on a truck or doll presented simultaneously in a puppet theater for two brief time intervals (10 s), counterbalancing for side of presentation. Across both trials, infant girls compared to boys directed more visual interest to the doll, whereas infant boys compared to girls directed more visual interest to the truck. These findings are clearly consistent with the hypothesized greater social sensitivity in females relative to males. They also imply that the well-documented emergence of gender-linked toy play in the second year of life (52–54) may build on inborn preferences in males and females for the perceptual features that define the conceptual categories of “masculine” and “feminine” toys.

**Figure 1:**
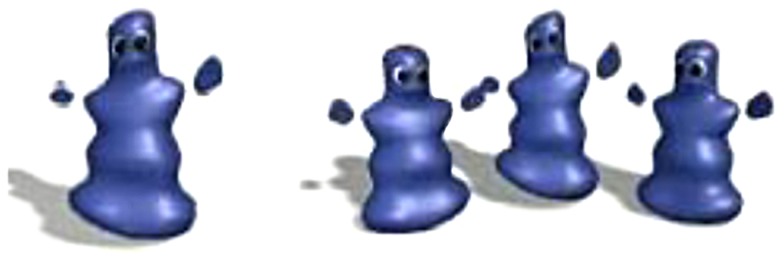
**Male infants with higher postnatal T show stronger visual preferences for the group of figures**.

In a second eye-tracking study of 41 infants at 3–4 months of age, we examined whether hormonal factors might contribute to early visual preferences by determining whether the ratio of the lengths of the second and fourth digits of the right hand (a putative marker of prenatal androgen action) (55) and salivary concentrations of T measured at 3–4 months of age would predict infants’ interest toward two categories of gender-linked stimuli: pictures of toys differentially preferred by older children (i.e., vehicle vs. doll) and a brief animation of a solitary figure and a group of figures (see Figure 1). The second set of stimuli were selected on the basis of previous research indicating innate sex differences in primate social organization, such that males compared to females prefer interactions with larger numbers of individuals (56). In male infants, smaller digit ratios suggestive of greater androgen action in prenatal life predicted greater visual attention to the male-typical toys, consistent with the earlier research indicating higher prenatal androgens result in stronger preferences for male-typical toys (57). Contrary to our predictions, salivary T concentrations measured in early postnatal life were unrelated to visual interest directed to gender-linked toys. However, whereas digit ratios were unrelated to interest directed to the animated figures, higher salivary T concentrations in male infants predicted greater visual attention to the group of figures. To our knowledge, this finding represents the first documented association between postnatal T and gender-linked preferences in infancy.

Interpretation of the association between higher salivary T in male infants and visual attention to a group of figures representing male-typical social organization may be informed by our other finding that higher salivary T in male infants at 3–4 months of age also predicted maternal ratings of greater negative affectivity on a well-established measure of infant temperament (58). Further analyses showed that the effect on negative affectivity was attributable to a strong association between higher T concentrations in male infants and infant frustration, as defined by fussing, crying, or showing distress when confined or unable to perform a desired action. Frustration in infancy is a temperament variable thought to contribute to the development of activity and aggression (59), personality traits with adaptive significance for males. Consistent with past speculation that a reduced independence from the mother following suppression of postnatal T may indicate neonatal T organizes the dynamics of primate social interactions (20), we hypothesize these recent findings from our research in human infants suggest postnatal T may be necessary for developmental programing of male social relations.

It is notable that although, we found associations between biological factors and gender-relevant behavior in our infant research, there were no sex differences in infant behavior at 3–4 months of age. For that reason, we describe behavior measured at 3–4 months of age as “pre-emergent” sex-linked behavior (8). We explain associations between digit ratios and visual attention to male-typical toys by suggesting that prenatal androgen concentrations may influence the incentive value of social stimuli in very early postnatal life and thereby promote the well-documented sex differences in object preferences in later life. Similarly, we suggest that postnatal androgens may encourage preferences for groups and associated male personality traits, such as higher rates of aggression and increased activity levels, and thereby influence the development of male-typical social structures.

Evidence of any organizational effects of postnatal T on behavior, however, requires establishing associations between postnatal concentrations of T and behavioral outcomes beyond the infantile period of transient HPG activation. For that reason, we tested whether postnatal T concentrations measured at 3–4 months of age would predict gender-typical play behavior and activity levels in children in the second year of life (60). Play preferences were videotaped and children’s interactions with male-typical, female-typical, and gender-neutral toys were coded later using behavioral coding software (Noldus Observer XT). In addition, children wore small accelerometers (i.e., actigraphs) that provided direct recording of movement during the play sessions. Actigraph data has been validated against other physiological measures of energy expenditure in children (61) and used widely as a measure of the cumulative intensity and frequency of movement in studies of activity related dysfunctions in childhood (e.g., ADHD) (62). Consistent with evidence from studies of girls with CAH, more male-typical digit ratios in girls predicted higher activity counts during play and weaker preferences for female-typical toys. In contrast, in both sexes, salivary T concentrations measured at 3–4 months of age were unrelated to our two measures of gender-linked behavior in young children.

Sex differences in social relations include verbal ability and emotional processing. Therefore, it is noteworthy that in contrast to the absence of associations between postnatal T concentrations and toddler toy choices summarized above, higher T concentrations at 3–4 months of age predicted higher scores on the autism spectrum disorder (ASD) scale of the Brief Infant-Toddler Social and Emotional Assessment (63) and lower verbalization in 84 children at 18 months of age (64). Postnatal T was unrelated to behavioral aggression and eye contact coded during toy play with the care-giver (65), which argues against our proposal that postnatal T may facilitate personality traits such as aggression. However, our measure of aggression was limited to behavior during toy play and a stronger test of the association between postnatal T and these aspects of sex-linked social behavior would come from an examination of children’s response to peers in an unstructured setting or levels of aggression following puberty.

Our findings in toddlers are consistent with those from our earlier research on infants. Associations between digit ratios and interest in gender-linked toys in the first and second year of life suggest prenatal androgens influence pre-emergent and emergent sex-linked object interests. Additionally, our findings of no association between T concentrations in the early postnatal period and toy interests suggest the postnatal developmental programing hypothesis may not extend to all aspects of gender-linked behavior. Findings from another lab (66) appear inconsistent with this conclusion. Urinary T concentrations collected seven times across the first 6 months of life correlated positively with parental reports of more male-typical preschool activities in boys but not girls at 14 months of age. The different results reported in this investigation may be attributable to the different hormone measurement. In addition, the parental report measure includes a range of gender-linked activities, including play with sex-typed toys, engagement in sex-typed activities (e.g., ballgames, cooking/cleaning play), and sex-typed child characteristics (e.g., interested in snakes, likes pretty things). Whether all three components contributed to the observed association between T in postnatal life and the global score on the parental report measure is unknown. Further, the observed correlations between T and children’s toy preferences are difficult to interpret. Boys with higher concentrations of postnatal T played less with a male-typical toy (a truck) and a female-typical toy (a baby doll) and more with a gender-neutral toy (soft book). Girls with higher concentrations of postnatal T played more with one male-typical toy (train) – but postnatal concentrations of T were unrelated to time girls spent playing with another male-typical toys (truck) or female-typical toys (tea set, dolls). The inconsistent behavioral data strengthen the possibility that aspects of the parental reports sensitive to postnatal T concentrations in boys are those involving activities that recruit social organization preferences (group vs. solitary) and are influenced by temperament.

## Future Directions

Our research indicating androgen concentrations in early postnatal life may influence risk for ASD is interesting given a need to better understand the mechanisms that contribute to the sex-biased prevalence rate and the deficits in communication and social interactions that characterize ASD. Indeed, ASD, a neurodevelopmental disorder diagnosed more frequently in boys, is associated with impairments in social interactions, restricted and stereotypical behaviors, and communication delays (67). Because typically developing boys relative to girls show decreased empathy and emotional processing (42), more frequent stereotyped interests (68), and decreased verbal abilities (69), behavioral features of ASD appear consistent with a “hypermasculinization” of the brain suggesting higher prenatal androgens in males may be a risk factor for the disorder (70). Consistent with this possibility, measures of higher prenatal T in typically developing children are associated with relative deficiencies in social interactions (e.g., eye contact and empathy) (71, 72) and language development (73). Girls exposed to higher prenatal androgens because of an endocrine disorder report more autistic traits than unaffected relatives (72) and digit ratios (a putative proxy for prenatal androgen action) (55) are smaller (i.e., more masculine) in children with ASD than unaffected, unrelated children (74). Our findings add to this literature by indicating a possible role for concentrations of T in the postnatal period. Interestingly, in a longitudinal investigation of over 800 children, umbilical cord blood concentrations of bioavailable T measured at birth were not associated with significant negative behavioral outcomes in either sex. Indeed, boys with higher concentrations of bioavailable T in cord blood at birth showed lower scores on a measure of attention problems at 5–10 years of age (75). In sum, these findings suggest that it may be informative in future studies of the postnatal programing hypothesis to investigate the possible interaction between antenatal and postnatal concentrations of T on positive and negative behavioral outcomes by including measurement of T concentrations in cord blood and during the postnatal surge at 1–3 months of age.

Finally, we have shown one measure of salivary T at 3–4 months of age can predict aspects of infant and toddler behavior. In this relatively uncharted research area, these findings clearly require independent replication. Nonetheless, the general results of this growing body of research support the earlier speculation (14) that the exaggerated rise in T secretion in preterm infants (15) or exposure to hormone disruptors in early postnatal life (16) may have adverse consequences for male primate reproductive development. Today, it is known that higher postnatal salivary T concentrations in very low birth weight babies are associated with greater health problems, including delays in growth and longer hospitalizations (76) and infants are widely exposed to substances in formula, food, breast-milk that can disrupt normal hormonal processes in postnatal life (77). Our research findings suggest that future research in these areas may inform understanding of hormone–behavior relations in early development by including measurement of gender-relevant behavioral outcomes.

## Conflict of Interest Statement

The author declares that the research was conducted in the absence of any commercial or financial relationships that could be construed as a potential conflict of interest.
